# Supplementary material for: LRRK2 suppresses lysosome degradative activity in macrophages and microglia through MiT-TFE transcription factor inhibition
Source: Proc Natl Acad Sci U S A. 2023 Jul 24;120(31):e2303789120. doi: 10.1073/pnas.2303789120 (PMC10400961; doi:10.1073/pnas.2303789120)
Supplement: Supplementary file 1 — Appendix 01 (PDF) [file pnas.2303789120.sapp.pdf]

PNAS

Supporting Information for

LRRK2 Suppresses Lysosome Degradative Activity in Macrophages and Microglia via MiT-TFE Transcription Factor Inhibition

Narayana Yadavalli[1,2,3,4,6], and Shawn M. Ferguson[1,2,3,4,5,6*]

Departments of Cell Biology[1] and Neuroscience[2], Program in Cellular Neuroscience, Neurodegeneration and Repair[3], Wu Tsai Institute[4], Kavli Institute for Neuroscience[5], Yale University School of Medicine, New Haven, Connecticut 06510, USA. Aligning Science Across Parkinson's (ASAP) Collaborative Research Network, Chevy Chase, MD, 20815, USA.[6]

*Correspondence: shawn.ferguson@yale.edu

This PDF file includes:

Supporting text
Figures S1 to S9
Table S1 and S2

Supplemental Figure 1

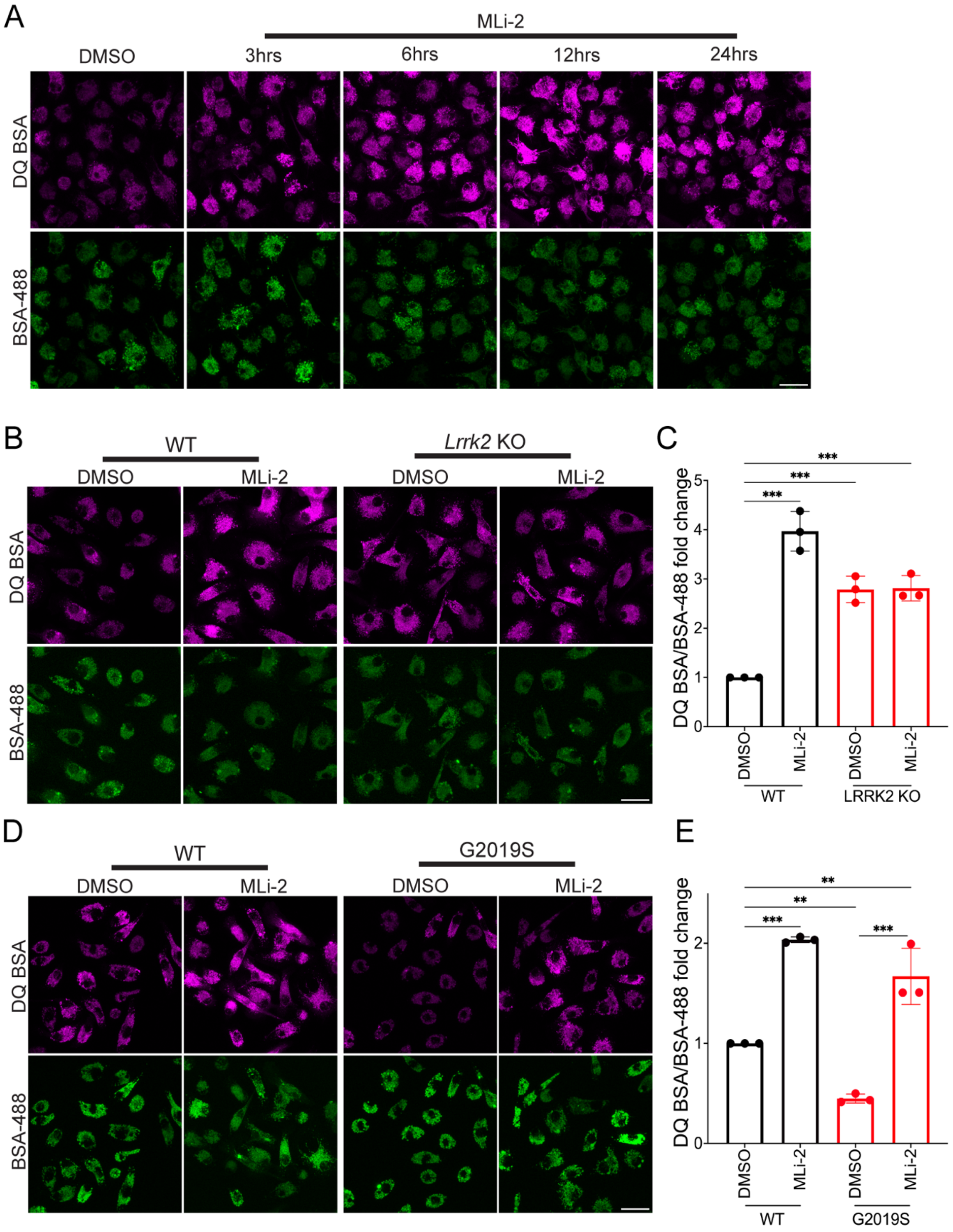

Figure S1: LRRK2 negatively regulates lysosomal proteolytic activity in mouse bone marrow derived macrophages (BMDMs). (A) Confocal micrographs showing the DQ-BSA and Alexa488-BSA fluorescence in WT cells treated with 0.1% DMSO or 50 nM MLi-2 in for 3,6,12 and 24 hours. Scale bar, 10 µm. **(B)** Confocal micrographs showing DQ-BSA and Alexa488-BSA fluorescence in WT and LRRK2 KO BMDMs treated for 3 hours with 0.1% DMSO or 50 nM MLi2. Scale bar, 10 µm. **(C)** Quantification of the DQ-BSA/Alexa488-BSA fluorescence. The mean fluorescence intensity of DQ-BSA was normalized to Alexa488-BSA and plotted relative to the DMSO control. The data was collected from 3 independent experiments with 60-80 cells per experiment. **(D)** Confocal micrographs showing DQ-BSA and Alexa488-BSA fluorescence in WT and LRRK2 G2019S knockin mouse BMDMs that were treated with 0.1% DMSO or 50 nM MLi2 for 3 hours. Scale bar, 10 µm. **(E)** Quantification of DQ-BSA/Alexa488-BSA fluorescence in WT and LRRK2 G2019S macrophages. The data was collected from 3 independent experiments with 60-80 cells per experiment (error bars show mean ± SEM, one-way ANOVA, * $p<0.05$, ** $p< 0.01$, ***$p< 0.001$).

Supplemental Figure 2

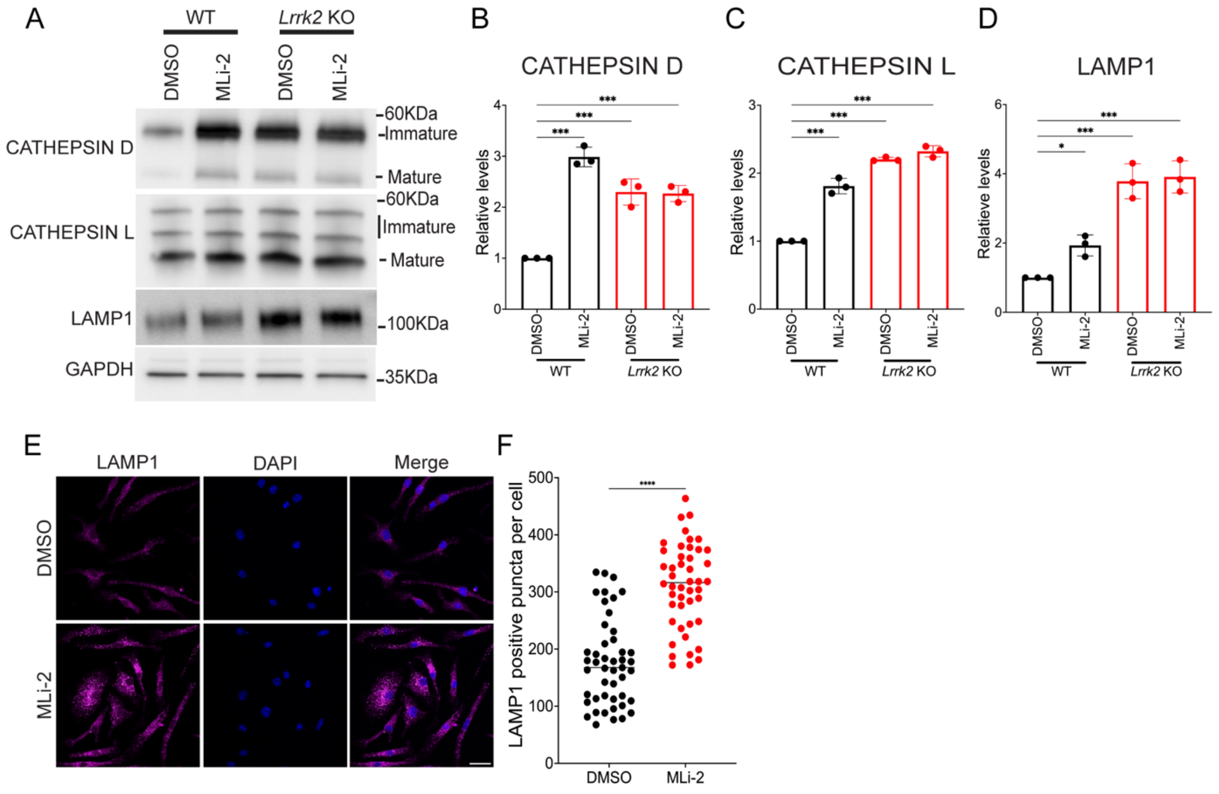

Figure S2: LRRK2 KO and MLi2 treatment increase the abundance of multiple lysosome proteins in mouse bone marrow derived macrophages. (A) Immunoblotting analysis of WT and LRRK2 KO mouse BMDMs treated with 0.1% DMSO or 50 nM MLi-2 for 6 hours. **(B-D)** Quantification of immunoblots in A. Data was plotted in relative to the DMSO-treated WT cells (n=3 independent experiments). **(E)** Confocal micrographs showing the LAMP1 immunostaining in mouse BMDMs treated with 0.1% DMSO or 50 nM MLi-2 for 6 hours. Scale bar, 10 μm. **(F)** Quantification of LAMP1 positive puncta shown in E (n=3 experiments with 17-20 cells analyzed per experiment. For all panels error bars show mean ± SEM, one-way ANOVA, * p<0.05, ** p< 0.01, *** p< 0.001).

Supplemental Figure 3

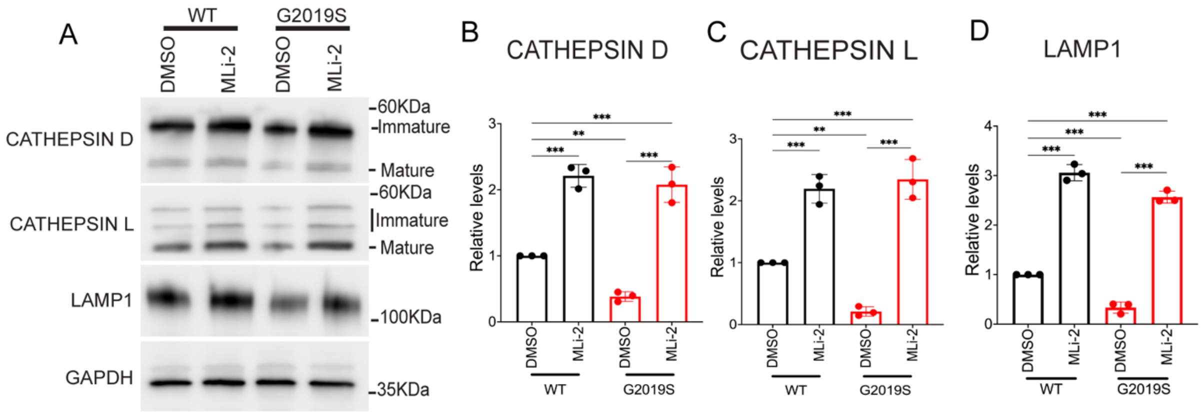

Figure S3: LRRK2 G2019S knockin mouse bone marrow derived macrophages have reduced levels of multiple lysosome proteins. (A) Immunoblot analysis of WT and LRRK2 G2019S mutant BMDM's treated with 0.1% DMSO or 50 nM MLi-2 for 6 hours. **(B-D)** Quantification of immunoblots from panel A. Data was plotted relative to the DMSO-treated WT cells (n=3, error bars show mean ± SEM, one-way ANOVA, * p<0.05, ** p< 0.01, *** p< 0.001).

Supplemental Figure 4

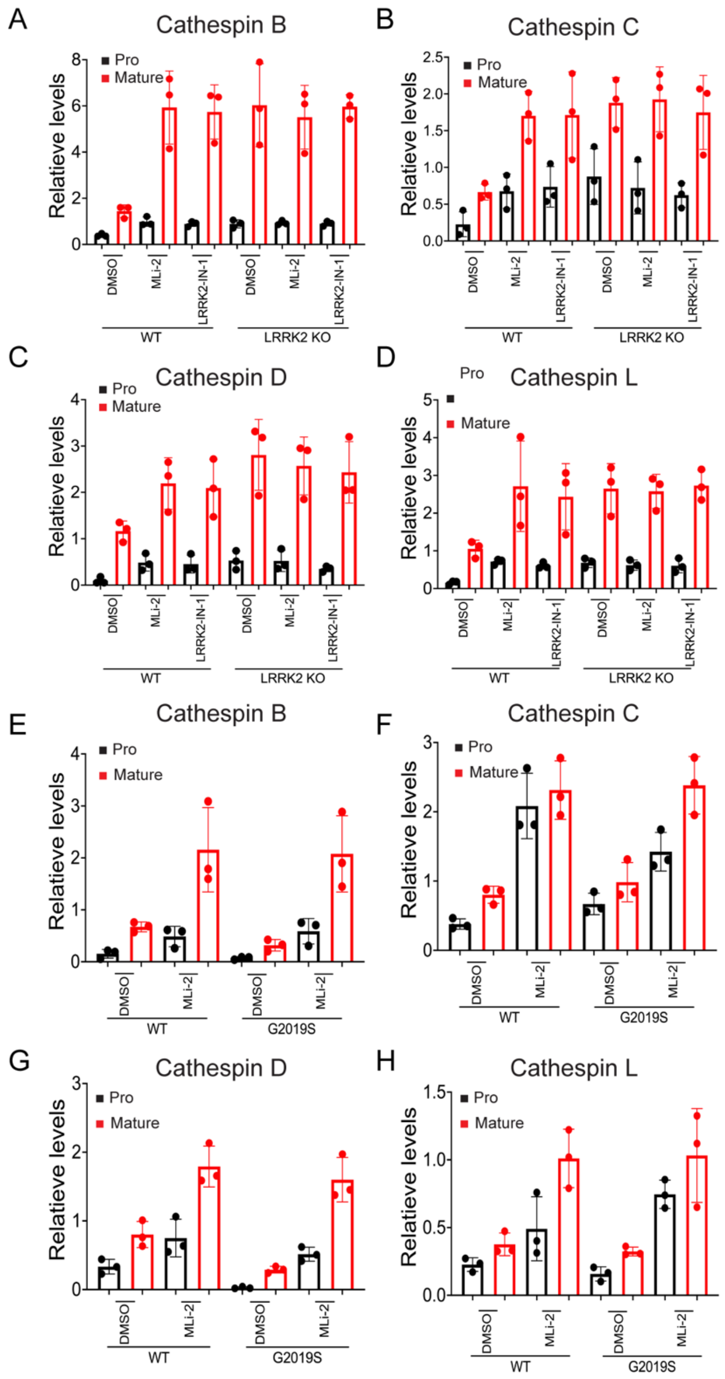

Figure S4: LRRK2 KO and MLi2 treatment increase the abundance of multiple lysosome proteins in human iPSC derived macrophages. (A-D) Bar graphs showing the quantification of immature and mature cathepsin B, C, D and L levels

in LRRK2 KO and MLi-2 treated conditions. This analysis was performed on the data presented in Figure 2. **(E-H)** Bar graphs showing the quantification of immature and mature cathepsin B, C, D and L levels in G2019S knockin and MLi-2 treated conditions (based on re-analysis of results from Figure 3).

Supplemental Figure 5

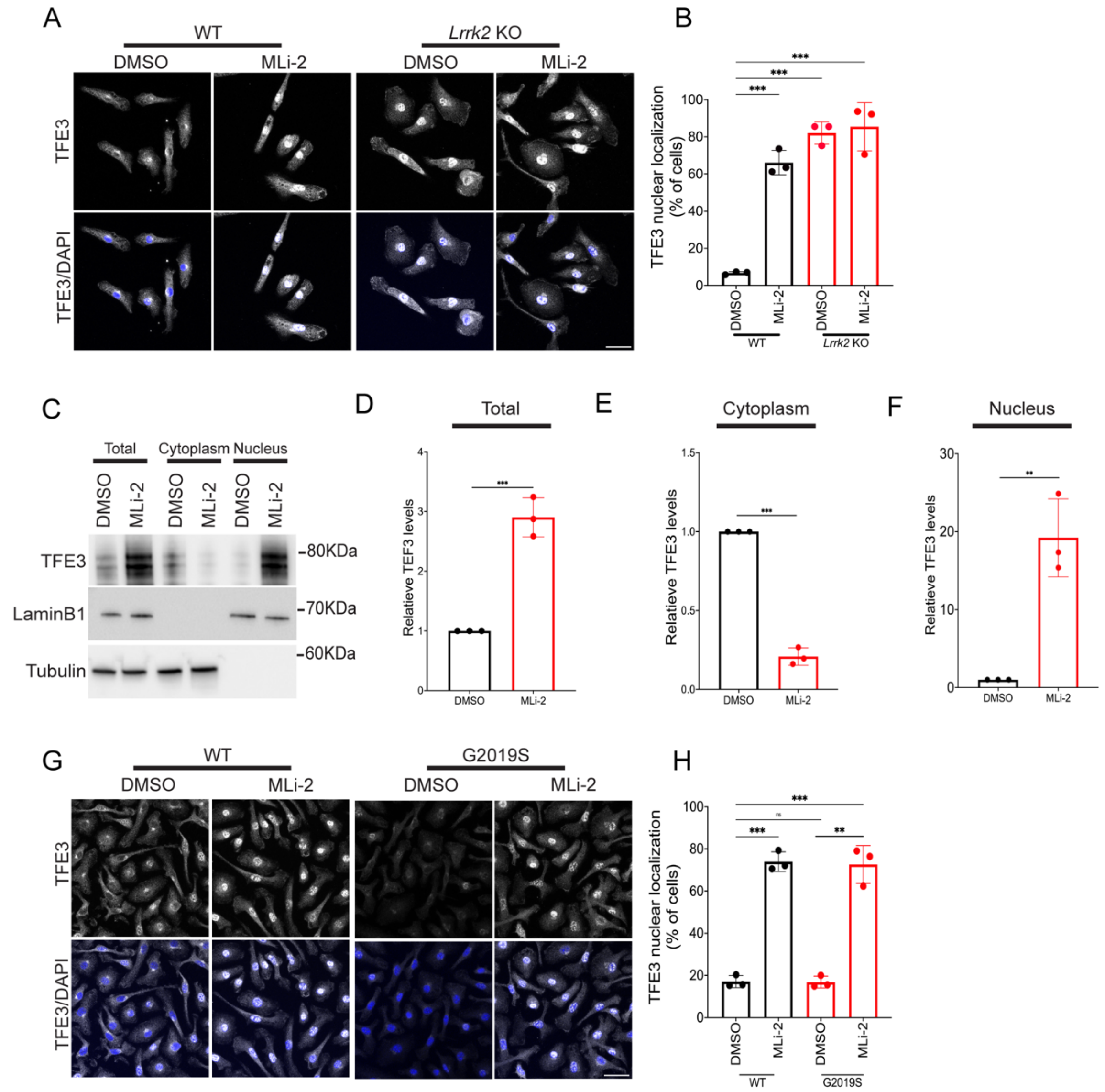

Figure S5: LRRK2 negatively regulates the nuclear localization of TFE3 in mouse bone marrow derived macrophages. (A) Immunofluorescence confocal micrographs showing the sub-cellular localization of TFE3 in WT and LRRK2 KO

BMDMs that were treated with 0.1% DMSO or 50 nM MLi-2 for 3 hours. Scale bar, 10 µm. **(B)** Quantification of the % of cells with nuclear>cytoplasmic TFE3. Data was collected from 3 independent experiments with approximately 50-60 cells analyzed per experiment. **(C)** Immunoblots showing the levels of TFE3 in total cell lysate, cytoplasmic and nuclear fractions from BMDM's treated with 0.1% DMSO or 50nM MLi-2 for 6 hours. **(D-F)** Quantification of immunoblots shown in C. **(G)** Immunofluorescence confocal micrographs showing the sub-cellular localization of TFE3 in WT and LRRK2 G2019 mutant BMDMs that were treated with 0.1% DMSO or 50 nM MLi-2 for 3 hours. Scale bar, 10 µm. **(H)** Quantification of the % cells with nuclear>cytoplasmic TFE3 under the indicated conditions. Data was collected from 3 independent experiments with approximately 50-60 cells per experiment. Scale bars = 10µm, error bars show mean ± SEM, error bars show mean ± SEM, one-way ANOVA, * p<0.05, ** p< 0.01, ***p< 0.001.

Supplemental Figure 6

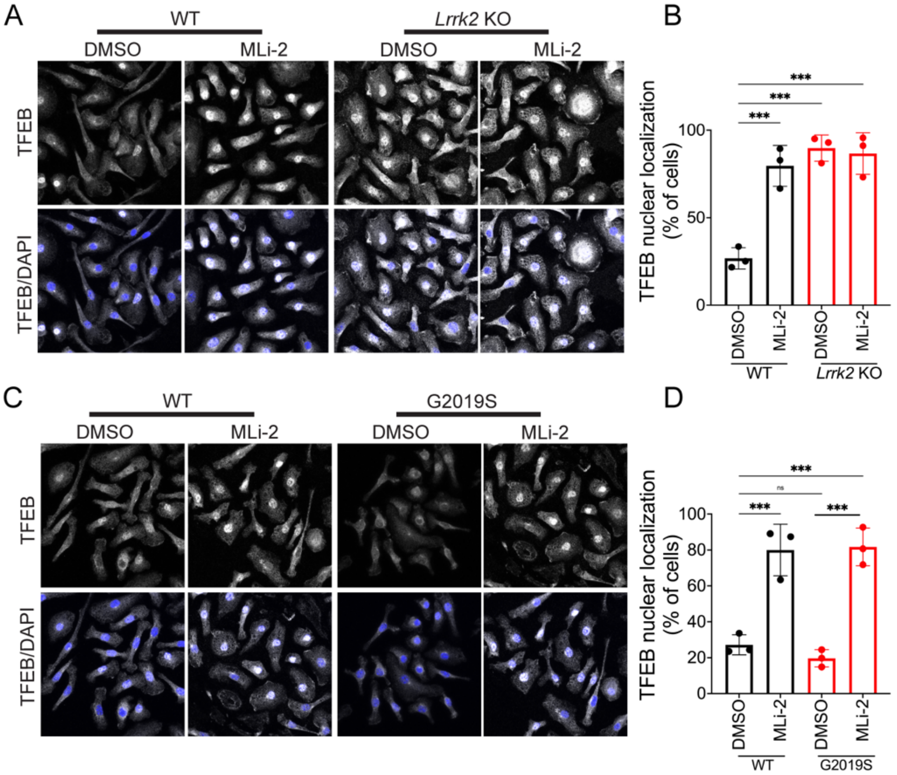

Figure S6: LRRK2 negatively regulates the nuclear localization of TFEB in mouse bone marrow derived macrophages. (A) Immunofluorescence, confocal micrographs showing the sub-cellular localization of TFEB in WT and LRRK2 KO BMDMs that were treated with 0.1% DMSO or 50 nM MLi-2 for 3 hours. Scale bar, 10 µm. **(B)** Quantification of the % of cells with nuclear>cytoplasmic TFEB. Data was collected from 3 independent experiments with approximately 50-60 cells analyzed per experiment. **(C)** Immunofluorescence, confocal micrographs showing the sub-cellular localization of TFEB in WT and LRRK2 G2019 mutant BMDMs that were treated with 0.1% DMSO or 50 nM MLi-2 for 3 hours. Scale bar, 10 µm. **(D)** Quantification of the % cells with nuclear>cytoplasmic TFEB under the indicated conditions. Data was collected from 3 independent experiments with approximately 50-60 cells per experiment. Scale bars = 10µm, error bars show mean ± SEM, one-way ANOVA, * $p<0.05$, ** $p< 0.01$, *** $p< 0.001$.

Supplemental Figure 7

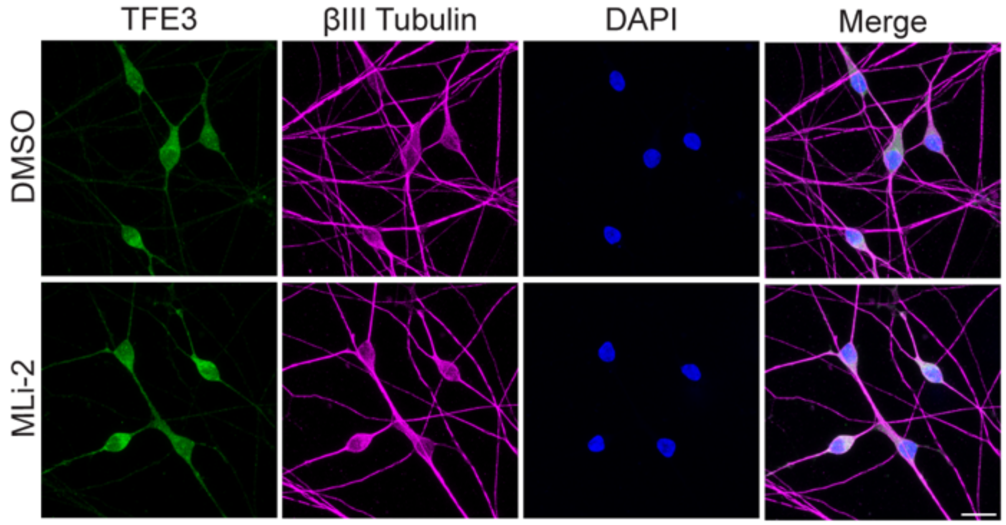

Figure S7. Confocal micrographs showing TFE3 localization in human iPSC derived cortical neurons treated with 0.1% DMSO or 50nM MLi-2 for 6 hours. Scale bar = 10 µm.

Supplemental Figure 8

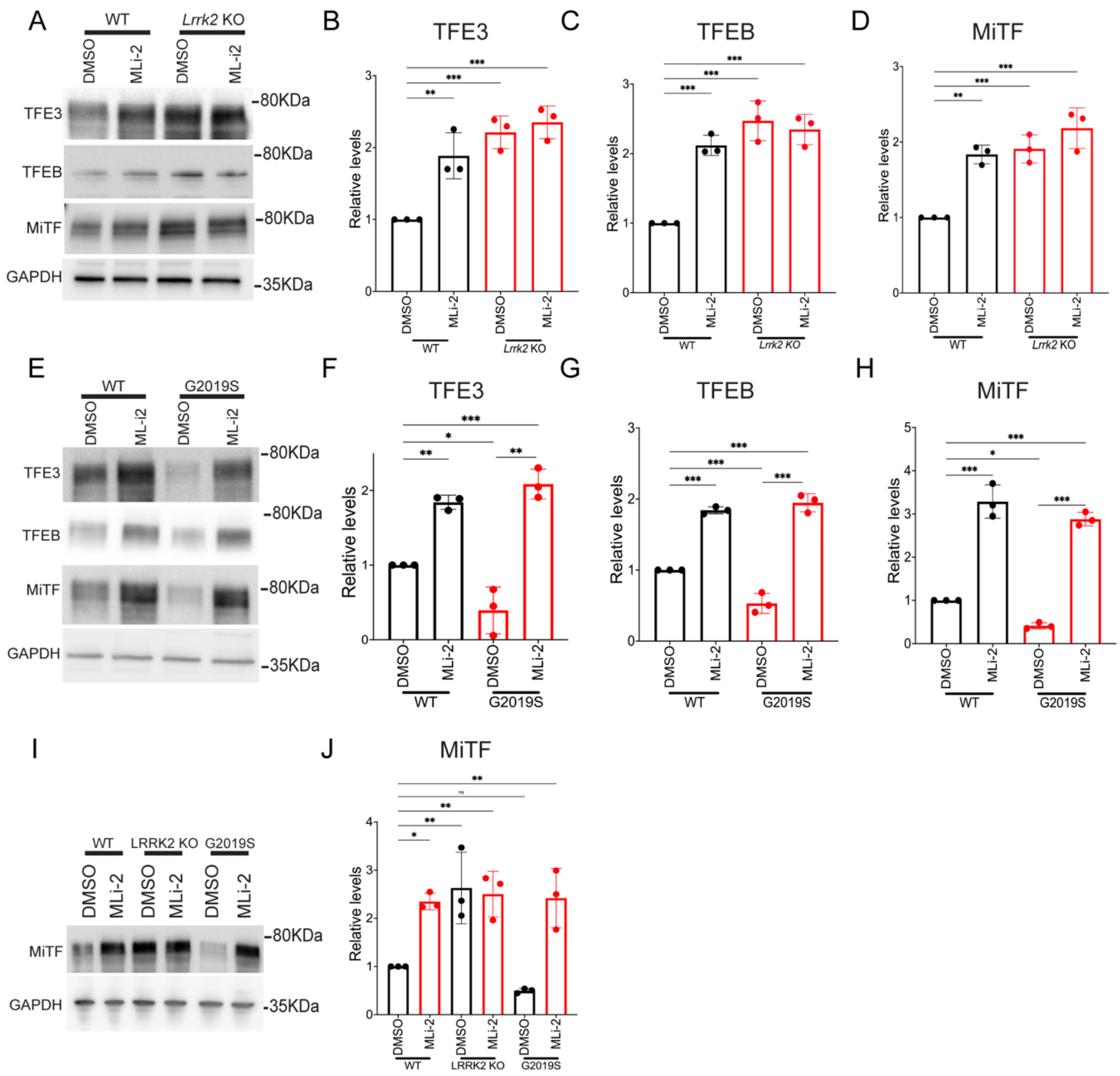

Figure S8: LRRK2 negatively regulates the abundance of TFE3, TFEB and MITF in mouse bone marrow derived macrophages. (A) Immunoblots showing the levels of TFE3, TFEB and MiTF in WT and LRRK2 KO cells treated with 0.1% DMSO or 50nM MLi-2 for 6 hours. **(B-D)** Quantification of immunoblots from panel A. Data was collected from 3 independent experiments, normalized to GAPDH and plotted relative to DMSO-treated WT cells. **(E)** Immunoblots showing the levels of TFE3, TFEB and MITF in WT and LRRK2 G2019S macrophages. **(F-H)** Quantification of immunoblots in panel E. Data was collected from 3 independent experiments, normalized to GAPDH and represented relative to the DMSO-treated

WT macrophages. **(I)** Immunoblots showing the levels of MITF in WT, LRRK2 KO and LRRK2 G2019S BMDMs cells treated with 0.1% DMSO or 50nM MLi-2 for 6 hours. **(J)** Quantification of immunoblots shown in M. Data was collected from 3 independent experiments, normalized to GAPDH and represented relative to the control siRNA treated WT macrophages. Error bars show mean ± SEM, one-way ANOVA, * p<0.05, ** p< 0.01, ***p< 0.001.

Supplemental Figure 9

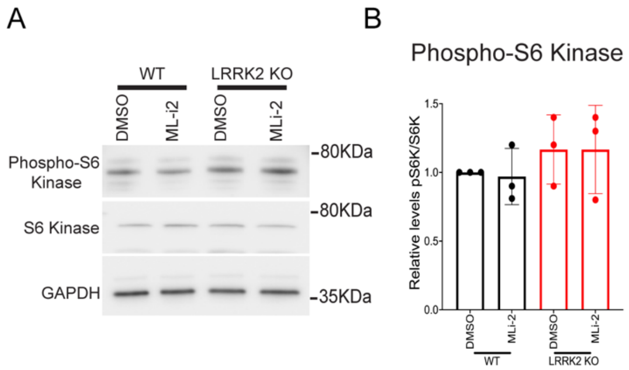

Figure S9: LRRK2 inhibition does not affect the mTORC1-dependent phosphorylation of ribosomal protein S6 kinase. (A) Immunoblots from human iPSC-derived macrophages showing the levels of phosphorylated S6 Kinase (T389), total S6 kinase and GAPDH in WT and LRRK2 KO cells treated with 0.1% DMSO or 50nM MLi-2 for 6 hours. **(B)** Quantification of immunoblots where data was collected from 3 independent experiments, normalized to GAPDH and plotted relative to DMSO-treated WT cells. Error bars show mean ± SEM.

Table S1: Sources of antibodies used.

Antibody	Company	Catalog Number	RRID
Human cathepsin B	R&D Systems	AF953	AB_355738
Human cathepsin C	R&D Systems	AF1071	AB_2086965
Human Cathepsin D	R&D Systems	AF1014	AB_2087218
Mouse Cathepsin D	R&D Systems	AF1029	AB_2087094
Human cathepsin L	R&D Systems	AF952	AB_355737
Mouse cathepsin L	R&D Systems	AF1515	AB_2087690
Human GBA	R&D Systems	MAB7410	AB_2938856
GAPDH	EnCor Biotechnology Inc	MCA-1D4	AB_2107599
Human LAMP1	Cell signaling Technology	9091	AB_2687579
Mouse LAMP1	DSHB	1D4B	AB_2134500
MITF	Cell signaling Technology	12590	AB_2616024
MITF	Abcam	Ab303530	AB_2938857
S6K	Cell Signaling Technology	9202	AB_331676
S6K-Phospho-T389	Cell Signaling Technology	9205	AB_330944
Human TFEB	Cell Signaling Technology	37785	AB_2799119
Mouse TFEB	Proteintech	13372-1-AP	AB_2199611
TFE3	Sigma	HPA023881	AB_1857931

Table S2. Oligonucleotide primer sequences used for qRT-PCR assays.

CTSL-FP	CTGGTGGTTGGCTACGGATT
CTSL-RP	CTCCGGTCTTTGGCCATCTT
CTSD-FP	AACTGCTGGACATCGCTTGCT
CTSD-RP	CATTCTTCACGTAGGTGCTGGA
TFE3-FP	ACTACTGTCAGCAACTCCTG
TFE3-RP	CTGTCGTTAACGTTGAATCGCC
GAPDH-FP	TGCACCACCAACTGCTTAGC
GAPDH-RP	GGCTAGGACTGTGGTCATGAG
LAMP1-FP	CCTGGGTGCCACTAACACAT
LAMP1-RP	CACACTTTTCCCCATAGCG
GBA-FP	ATGCAAGAGTGAATGGGAAGG
GBA-RP	TTCATTCTCCGCTGTCACTC
